# Iatrogenic PVD Following Dilated Fundus Examination: A New Diagnosis or Fluke?

**DOI:** 10.18502/jovr.v17i1.10182

**Published:** 2022-01-21

**Authors:** Patrick W Commiskey, Gagan Kalra, Jay Chhablani

**Affiliations:** ^1^University of Pittsburgh Medical Center, Pittsburgh, PA, USA; ^2^Government Medical College and Hospital, Chandigarh, India

##  PRESENTATION

**Figure 1:**
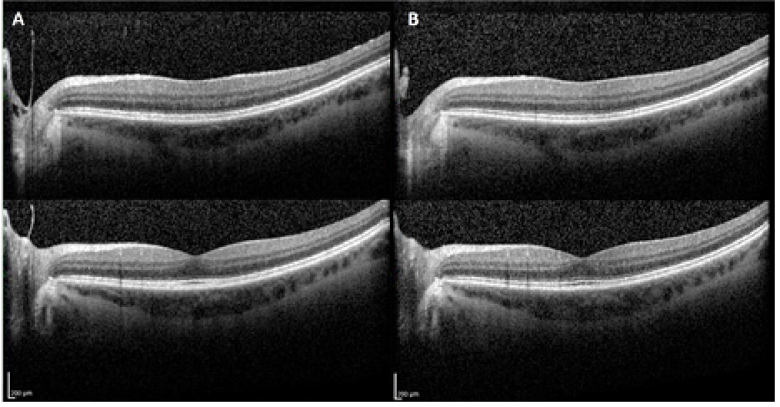
(A) Two optical coherence tomography (OCT) images at the initial visit for a dilated eye exam showed posterior vitreous still attached at the disc margin. (B)Two OCT images at the follow-up visit with new floaters due to posterior vitreous detachment (PVD) 2 hr following the initial exam.

A 55-year-old woman with high myopia (spherical equivalent –8.25 right eye [OD], –8.50 left eye [OS]) and known history of Sjogren syndrome presented for routine asymptomatic hydroxychloroquine screening. No other relevant past ocular, medical, or family history was noted. Visual acuity was found to be 20/20 in both eyes (OU). Dilated fundus examination (DFE) without indentation was performed using tropicamide and phenylephrine, and ancillary testing was performed. No signs of hydroxychloroquine toxicity, Weiss ring, retinal tears, or peripheral retinal pathology were noted OU. Incidentally, the screening spectral domain optical coherence tomography (SD-OCT) revealed tenuous vitreous attachment to the optic disc OS [Figure 1A].

Two hours after the screening outpatient examination, the patient experienced a new floater OS for which she subsequently presented to the ophthalmology emergency. The on-call ophthalmologist noted a Weiss ring OS without retinal tears or detachments OU. A subsequent SD-OCT scan in the left eye demonstrated a complete posterior vitreous detachment (PVD) [Figure 1B]. The patient was reassured, counselled about the warning symptoms of retinal detachment, and scheduled for a repeat DFE in one week.

##  DISCUSSION

To the best of our knowledge, this is the first report of completion of an impending PVD following cycloplegic DFE. Accommodation-induced ciliary body contraction, anterior displacement of the lens–iris diaphragm, and axial elongation of the vitreous cavity may be more pronounced in myopic patients.^[1]^ There is a known anatomical relationship between ciliary body contraction and anterior movement of anterior vitreous body and zonules.^[2,3]^ Anterior zonule movement results in adjacent anterior movement of the vitreous parallel to the sclera at the ora serrata.^[4]^ While it is unclear if this force would translate to posterior vitreous movement, there is an interesting case report of vitreo-macular traction (VMT) following pilocarpine-induced miosis after mydriasis.^[5]^ In a computer animation-based model of accommodation, it was found that during accommodation the vitreous base is pulled forward and the posterior vitreous is pushed backward.^[6]^ Axially, it is during the relaxation of accommodation that the posterior lens capsule moves anteriorly followed by subsequent anterior movement of posterior vitreous causing shearing at the retinal vitreous attachments.^[6]^ Although our report illustrates temporal association of completion of impending PVD following DFE, the precise changes in the posterior hyaloid in response to DFE are still unclear.

Further work could be done to elucidate the relationship between accommodative state and PVD. Wide-field OCT may expand our understanding of peripheral vitreoretinal physiology.

##  Financial Support and Sponsorship

Nil.

##  Conflicts of Interest

There are no conflicts of interest.
